# CRISPR/Cas9 gene editing: a new approach for overcoming drug resistance in cancer

**DOI:** 10.1186/s11658-022-00348-2

**Published:** 2022-06-17

**Authors:** Mostafa Vaghari-Tabari, Parisa Hassanpour, Fatemeh Sadeghsoltani, Faezeh Malakoti, Forough Alemi, Durdi Qujeq, Zatollah Asemi, Bahman Yousefi

**Affiliations:** 1 Department of Clinical Biochemistry and Laboratory Medicine, School of Medicine, Tabriz University of Medical Sciences, Tabriz, Iran; 2 Cellular and Molecular Biology Research Center (CMBRC), Health Research Institute, Babol University of Medical Sciences, Babol, Iran; 3 Department of Clinical Biochemistry, Babol University of Medical Sciences, Babol, Iran; 4 Research Center for Biochemistry and Nutrition in Metabolic Diseases, Kashan University of Medical Sciences, Kashan, Iran; 5 Molecular Medicine Research Center, Tabriz University of Medical Sciences, Tabriz, Iran

**Keywords:** CRISPR/Cas9, Gene editing, Chemoresistance, Malignancy, Cancer treatment

## Abstract

The CRISPR/Cas9 system is an RNA-based adaptive immune system in bacteria and archaea. Various studies have shown that it is possible to target a wide range of human genes and treat some human diseases, including cancers, by the CRISPR/Cas9 system. In fact, CRISPR/Cas9 gene editing is one of the most efficient genome manipulation techniques. Studies have shown that CRISPR/Cas9 technology, in addition to having the potential to be used as a new therapeutic approach in the treatment of cancers, can also be used to enhance the effectiveness of existing treatments. Undoubtedly, the issue of drug resistance is one of the main obstacles in the treatment of cancers. Cancer cells resist anticancer drugs by a variety of mechanisms, such as enhancing anticancer drugs efflux, enhancing DNA repair, enhancing stemness, and attenuating apoptosis. Mutations in some proteins of different cellular signaling pathways are associated with these events and drug resistance. Recent studies have shown that the CRISPR/Cas9 technique can be used to target important genes involved in these mechanisms, thereby increasing the effectiveness of anticancer drugs. In this review article, studies related to the applications of this technique in overcoming drug resistance in cancer cells will be reviewed. In addition, we will give a brief overview of the limitations of the CRISP/Cas9 gene-editing technique.

## Introduction

Cancer treatment is one of the most important health challenges. At present, surgery, chemotherapy, targeted therapy, and radiotherapy are among the routine approaches for cancer treatment in the clinic, depending on the patient’s condition [1–3]. However, cancers are still one of the leading causes of death. In 2020, cancer, with a total of 598,932 deaths, will continue to be the second leading cause of death in the USA after cardiovascular disease [4]. These statistics justify the need for further studies in the field of cancer treatment. In general, current research pursues two main goals: first, to find new ways to treat cancer, and second, to strengthen the effectiveness of current treatment approaches. One of the new approaches proposed for the treatment of cancers is genome manipulation. In this regard, the emerging CRISPR/Cas9 gene-editing technique has attracted a lot of attention. Using this technique, it is possible to manipulate the genome in cancer cells. The results of studies on cellular and animal models have shown that the CRISPR/Cas9 technique can be effective in treating cancers. Several clinical trials are also underway to evaluate the efficiency of CRISPR/Cas9 technology in treating cancers [5, 6]. On the other hand, studies have shown that this technique can also be used to enhance the effectiveness of chemotherapy and targeted therapy. One of the main problems in the treatment of cancers is the issue of drug resistance, which is estimated to cause 90% of deaths in patients with cancer [7]. Although many aspects of the mechanism of drug resistance in cancer cells are not yet known, current findings suggest that a significant number of genes associated with drug efflux, DNA repair, apoptosis, and various cellular signaling pathways are involved in drug resistance [8, 9]. Targeting some of these genes using CRISPR/Cas9 technology has yielded promising results in weakening drug resistance and increasing the effectiveness of anticancer drugs. In this review article, after an overview of the important mechanisms of drug resistance and some approaches that have been studied to inhibit drug resistance in cancer, we discuss the applications of the CRISPR/Cas9 technique to overcome drug resistance in cancers. In addition, limitations of the CRISPR/Cas9 technique are briefly discussed.

## Drug resistance: a major barrier in cancer treatment

Chemotherapy is a common approach in the treatment of cancers. However, cancer cells often have the ability to resist the effects of chemotherapeutic drugs, which can lead to chemotherapy failure [10]. In addition to conventional chemotherapy, other strategies have been developed to target key features and capabilities of malignant cells. Targeted therapy is a therapeutic strategy that has made significant advances in cancer treatment by disrupting these key features. In fact, targeted therapy is the result of advances in understanding the molecular mechanism of cancers, leading to highly effective therapies against tyrosine kinases and other molecular targets [11, 12]. EGFR in lung cancer [13], BRAF in melanoma [14], BCR–ABL fusion in chronic myeloid leukemia [15], HER2 in breast cancer [16], and FGFR in different types of cancer [17] are among the most important targets, and some well-known anticancer drugs have been designed against them. Recently, oncological treatment using immunological approaches has also advanced to detect and treat cancers [18], but resistance against targeted therapy and immunological therapy is still a common issue [19]. The ability of cancer cells to resist anticancer drugs, which can lead to enhanced survival, is called drug resistance. In the treatment of cancer, drug resistance is a major problem. Drug resistance can be divided into intrinsic (or de novo) resistance or acquired resistance. In intrinsic drug resistance, resistance factors are existed in tumor cells before drug treatment, while in acquired drug resistance, resistance can develop during drug treatment [20, 21]. An important feature of drug resistance is that resistance to one drug may lead to resistance to other drugs, which is called multidrug resistance (MDR). Cancer multidrug resistance is defined as the cross-resistance or insensitivity of cancer cells to the cytostatic or cytotoxic activity of various anticancer drugs. These drugs may differ in structure or function as well as molecular targets [22, 23]. Two main groups of factors are involved in creating drug resistance. The first group includes pharmacological and physiological factors, and the second group includes cell-/tissue-specific factors [24, 25]. Increasing the activity of drug efflux pumps such as ATP-binding cassette (ABC), drug detoxification enhancement, DNA repair ability enhancement, epigenetic effects, disruption in cellular signaling pathways, attenuation of apoptosis, and epithelial–mesenchymal transition (EMT) enhancement are among the most important of these mechanisms [22, 26–29]. Each of these mechanisms, independently or in combination, reduces the therapeutic effect of prescribed drugs and makes cancer treatment more difficult.

For example, a study by Karin Klappe et al. showed that multidrug resistance protein 1 (MRP1 or ABCC1) was overexpressed in HT29 colon cancer cells during the development of acquired drug resistance caused by colchicine [30]. Another study observed that epigenetic silencing of Spalt‐like transcription factor 2 (SALL2) leads to resistance against tamoxifen in breast cancer [31]. Studies suggest that failure to regulate signaling pathways, including YAP/TAZ signaling, may be among the main mechanisms of intrinsic and acquired resistance against chemotherapy and target therapy. YAP induces survival of pancreatic and colon cancer cells by enhancing EMT [32]. A study by Arumugam et al. found that EMT leads to drug resistance in pancreatic cancer. This resistance is exerted by an EMT regulator called Zeb1 [33]. A significant number of studies in the field of cancer treatment have been devoted to the purpose of targeting some of the above mechanisms to inhibit MDR in cancers, which will be reviewed in the following sections.

## Advances in the study of drug resistance inhibition to enhance chemosensitivity in cancer

So far, several approaches have been proposed to overcome drug resistance in cancer, and in recent years, studies in this field have accelerated. These studies have often focused on the efficacy of different chemical and natural compounds and targeting some of the above-mentioned mechanisms and their related cellular signaling pathways and long noncoding RNAs as well as miRNAs in drug-resistant cancer cell lines. Before briefly reviewing some of the most interesting findings of these studies, a brief description of cellular and animal models of drug resistance is helpful. There are three major protocols for creating a resistant cell line that is dose-dependent and time-dependent. In the first method to develop a drug-resistant cancer cell, a sensitive cell line is exposed to the drug, and the drug concentration increases stepwise. In the second method, sensitive cells are exposed to a constant concentration of the drug continuously and for a long period. In the third method, sensitive cells are exposed to repetitive cycles of treatment. For example, cells are treated with anticancer drugs for 3 days and then treated with a control medium for 7 days, and this process is repeated in the same way [34]. MTT test, estimating IC_50_, and calculating the resistance factor is routine in the process for assessing cell resistance. Evaluation of P-glycoprotein (p-gp) expression can also be useful and is usually done to identify resistant cells. The technical details are very well explained elsewhere [35, 36].

Given that the increased expression of ABC family transporters is a very important cause of MDR in cancer cells [37], transfection of mdr1-expressing vectors (such as plasmid containing mdr1 cDNA) encoding p.gp into cancer cells is also applicable in the development of drug-resistant cancer cells [38]. Animal models of MDR are also used in the study of drug resistance mechanisms. MDR mouse models can be generated by subcutaneous injection of established drug-resistant cancer cells into mice. After the injection, the tumors are allowed to grow and the volume of the tumors is monitored. After the volume of the tumors reaches a certain level, treatment with chemotherapy drugs is started. Finally, after a certain period, the mice are killed and their tumor tissue is used for the study [35]. These models are a significant aid to the study of approaches to overcoming drug resistance in cancer cells. As mentioned above, increasing the expression of ABC family transporters can reduce the concentration of drugs inside the cancer cell and cause drug resistance. A considerable number of recent studies have focused on approaches to undermining the performance of the ABC family transporters, which play a key role in MDR. A number of inhibitors of ABC family pumps have been suggested as helpful compounds for attenuating MDR in cancer. NSC23925, NSC77037, and curcumin are among these compounds. For example, YS-7a, a quinoline compound derived from NSC23925, can significantly attenuate p.gp transport function and have positive effects on reversing MDR in cancer cells [39]. A study on osteosarcoma showed that NSC-77037 could reverse ABCB1/ABCC1-dependent drug resistance and improve the efficacy of doxorubicin [40], indicating the usefulness of inhibiting ABCB1/ABCC1 using NSC-77037 in overcoming MDR in cancer. Curcumin is a natural compound that has been extensively studied to overcome MDR in cancers because it appears to be nontoxic even in high concentrations [34]. For example, a study on colorectal cancer cells showed that curcumin could inhibit p.gp transport activity and increase the intracellular accumulation of doxorubicin in drug-resistant cancer cells. The results of this study showed that curcumin has a significant effect in reversing p.gp-dependent drug resistance and potentiating the cytotoxic and apoptotic effects of doxorubicin [41]. The effect of metformin, an antidiabetic drug, on the drug resistance of cancer cells has also been considered in recent years. It seems that metformin can attenuate p.gp activity in doxorubicin-resistant cancer cells in a dose-dependent manner and potentiate the apoptotic effects of doxorubicin [42]. In fact, the use of some common drugs in the treatment of other diseases, such as metformin, may be a useful approach to reduce the activity of ABC family transporters and overcome MDR in cancers.

Because a significant number of chemotherapeutic drugs can enhance cell death through induction of DNA damage, DNA damage response and enhancement of DNA repair mechanisms are key mechanisms of drug resistance in cancer cells [43]. Targeting the DNA damage response and attenuating DNA repair using various compounds has also been an approach that has been widely studied in recent years to overcome drug resistance in cancers. For example, a study of cisplatin-resistant gastric cancer cells showed that PARP1 inhibitors (AG14361 and BYK204165) could potentiate the effects of cisplatin in inducing DNA damage and apoptosis in drug-resistant gastric cancer cells. The findings of this study showed that inhibition of PARP1 could suppress the stability of DNA-PKcs and attenuate the NHEJ repair system [44]. However, resistance to PARP inhibitors can also be a serious challenge, and effective solutions should be found to reduce this problem. For example, it seems that targeting neuropilin 1 using miR-200c could weaken the resistance of ovarian cancer cells to olaparib, a PARP inhibitor [45, 46]. It should be noted that PARP1 acts as a DNA damage sensor and plays a very important role in DNA repair [47]. It seems that melatonin can also weaken drug resistance in cancers by weakening DNA repair, and combining melatonin with chemotherapy drugs could be another approach to overcoming drug resistance in cancers [48] that deserves to be further studied. A study of hepatocellular carcinoma cells showed that melatonin could attenuate DNA repair by inducing long noncoding RNA RAD51-AS1 RNA and potentiate the cytotoxic effects of chemotherapy drugs. Long noncoding RNA RAD51-AS1 attenuates DNA repair by inhibiting RAD51 translation, which is a key factor in DNA repair [49]. In the following, we will discuss the role of noncoding RNAs in drug resistance. Further details regarding the role of DNA repair in the failure of cancer chemotherapy have also been extensively described elsewhere [50]. Inhibition of some signaling pathways using different inhibitors has also had positive results in attenuating drug resistance in cancer cells. For example, inhibition of Nrf2 with trigonelline, an alkaloid compound has been suggested as a useful approach to overcome oxaliplatin resistance in colorectal cancer cells [51]. Inhibition of the PI3K/AKT signaling pathway can also be useful in enhancing chemosensitivity [52]. Weakening of stemness by using different compounds is also useful in weakening drug resistance in cancers. One study showed that bufalin, a traditional medicine compound, could weaken the stemness and cisplatin resistance in colorectal cancer cells [53].

In addition, the design of nanoparticles for effective delivery of anticancer compounds and overcoming drug resistance in cancers has been one of the most interesting areas of research in recent years, and the results reported so far have been very interesting. For example, codelivery of metformin and doxorubicin in the form of PLGA–TPGS nanoparticles can enhance drug cellular uptake and attenuate drug efflux in DOX-resistant breast cancer cells, leading to enhanced cytotoxicity and apoptosis and attenuated drug in breast cancer cells [54]. Further details on the application of nanotechnology in overcoming drug resistance in cancers are discussed elsewhere [55]. Targeting the inflammatory process with the use of some routine drugs and antibodies may also be a useful approach in weakening the drug resistance of cancer cells. One study showed that aspirin, a conventional nonsteroidal antiinflammatory drug, could potentiate the apoptotic effects of cisplatin on cisplatin-resistant lung cancer cells [56]. MEDI5117, an anti-IL6 antibody, has also shown positive effects in enhancing chemosensitivity in several types of cancer cells [57]. One of the most interesting and promising approaches to overcome drug resistance in cancers is to focus on noncoding RNAs, especially miRNAs and lncRNAs, as therapeutic targets. lncRNAs are a class of noncoding RNA molecules that are more than 200 nucleotides in length [58]. Various studies have shown that these molecules are associated with key mechanisms of drug resistance in cancer cells. For example, it appears that lncRNA H19 could attenuate p.gp expression in doxorubicin-resistant hepatocellular carcinoma cells [59]. One study also showed that lncRNA-MALAT1 could upregulate ABCC1 and ABCB1 in cisplatin-resistant non-small-cell lung cancer cells, possibly through STAT3 activation [60]. Besides, it appears that this lncRNA could potentiate doxorubicin resistance in breast cancer cells by targeting miR-570-3p, while upregulating miR-570-3p could increase chemosensitivity in breast cancer cells [61]. lncRNA NEAT1 may be involved in enhancing DNA repair, so inhibition of this lncRNA causes downregulation of some genes involved in the homologous recombination pathway. Inhibition of this lncRNA can enhance the sensitivity of multiple myeloma cells to anticancer drugs, including bortezomib, carfilzomib, and melphalan [62]. Suppression of lncRNA LINC00963 has also been suggested as a useful solution to weaken stemness and drug resistance in oral cancer cells [63]. In view of the above, it seems that inhibiting some lncRNAs by antisense oligonucleotides or overexpressing some lncRNAs are useful and promising approaches to overcome drug resistance in cancers. miRNAs are another important class of noncoding RNA molecules and have a length of about 22 nucleotides [64].

By targeting mRNA, these molecules cause degradation, destabilization, and inhibition of translation, thus playing a role in posttranscriptional regulation [65–67]. In recent years, the design of anticancer drugs based on miRNA mimic and antagomiR has received much attention, and some of these drugs are in phase I clinical trial. For example, cobomarsen, an antagomiR-155, has been proposed as a useful drug in the treatment of cutaneous T-cell lymphoma and is being studied in phase I clinical trials [68]. In addition to lncRNAs, extensive studies have been performed on miRNAs as therapeutic targets for overcoming drug resistance in cancers. Some of these studies have reported promising results, and it is hoped that miRNA mimics and antagomiRs can be used to overcome drug resistance in cancers. One of these studies on doxorubicin-resistant osteosarcoma cells showed that the miR-221 inhibitor could significantly reduce the expression of p.gp and bcl-2, an anti-apoptotic protein, and attenuate drug resistance [69]. A study on ovarian cancer cells showed that miR-21 can also upregulate p.gp and play a role in cisplatin resistance [70]. It should be noted that miR-21 is a well-known oncomiR and is involved in drug resistance in a wide variety of cancers [71]. Inhibition of this miRNA is being seriously studied as an approach to overcome drug resistance in cancers. Besides, increased expression of miR-506 in oxaliplatin-resistant colorectal cancer cells may attenuate MDR1/p.gp expression and enhance chemosensitivity [72]. Therefore, it seems that miR-506 mimics can be further studied to overcome MDR in cancer cells. One study showed that miR-140 could attenuate DNA repair by inhibiting flap endonuclease 1 (FEN1), and increased expression of this miRNA could enhance chemosensitivity in breast cancer cells [73]. miR-200c can also reverse drug resistance in gastric cancer cells by downregulating ERCC3 and ERCC4, which may play a role in the nucleotide excision repair (NER) system [74]. miR451-a can attenuate EMT in lung cancer cells and increase sensitivity to doxorubicin by downregulating c-Myc. Overexpression of this miRNA also appears to have positive effects in potentiating the anticancer effects of doxorubicin in vivo [75]. These results suggest that the design of drugs based on miR-451a mimics may be a useful approach in weakening the drug resistance of cancers. It seems that miR-372 and miR-373 can enhance stemness and resistance to 5-FU in colorectal cancer cells [76]. Therefore, effective inhibition of these miRNAs may be a helpful approach to attenuate stemness and overcome drug resistance. The results of a study showed that miR-144 mimics could potentiate the cytotoxic and apoptotic effects of 5-FU in hepatocellular carcinoma cells, possibly by enhancing Nrf2 degradation [77].

**Fig. 1:**
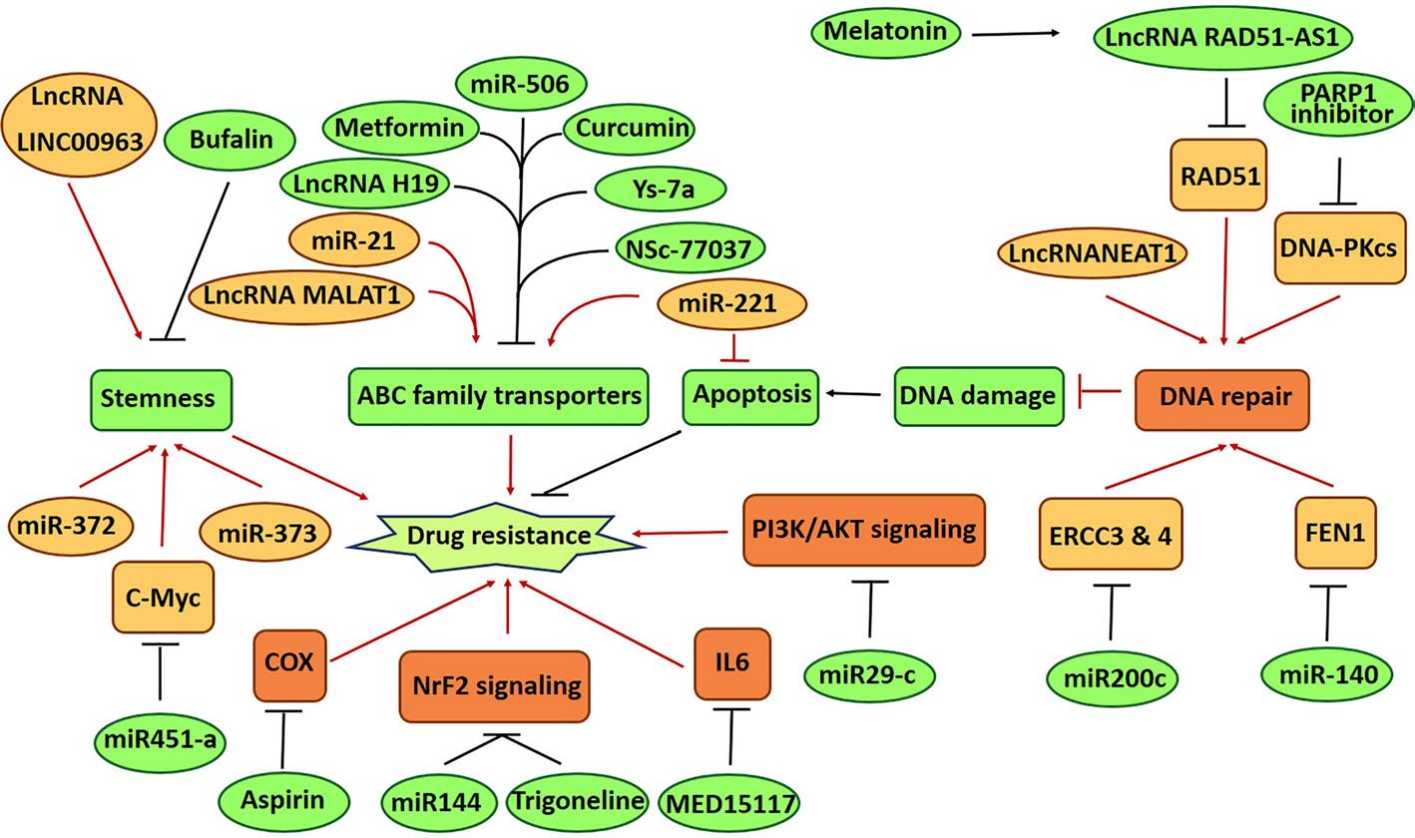
A summary of new advances in overcoming drug resistance in cancers

Another study showed that miR-29c may potentiate cisplatin sensitivity in non-small-cell lung cancer cells through negative regulation of PI3K/AKT signaling [78]. It should be noted that the association of miRNAs with signaling pathways is a very broad issue and not limited to Nrf2 and PI3K/AKT signaling. These issues have been discussed in detail elsewhere [27, 79]. Figure 1 summarizes some of the important findings reviewed in this section. In recent years and with the development of genome editing techniques, there is hope that it may be possible to overcome the drug resistance barrier of cancers by manipulating the genome and targeting the key mechanisms of drug resistance that are mentioned above and improve the effectiveness of anticancer drugs. As noted in the introduction, CRISPR/Cas9 gene editing is one of the most successful genome editing techniques. In recent years, some studies have shown that targeting some factors involved in drug resistance using this technique can significantly enhance the effectiveness of anticancer drugs. In the following, the applications of CRISPR/Cas9 gene editing in overcoming drug resistance in cancers will be discussed.

## CRISPR/Cas9 gene editing applications in overcoming drug resistance in cancers

**Fig. 2:**
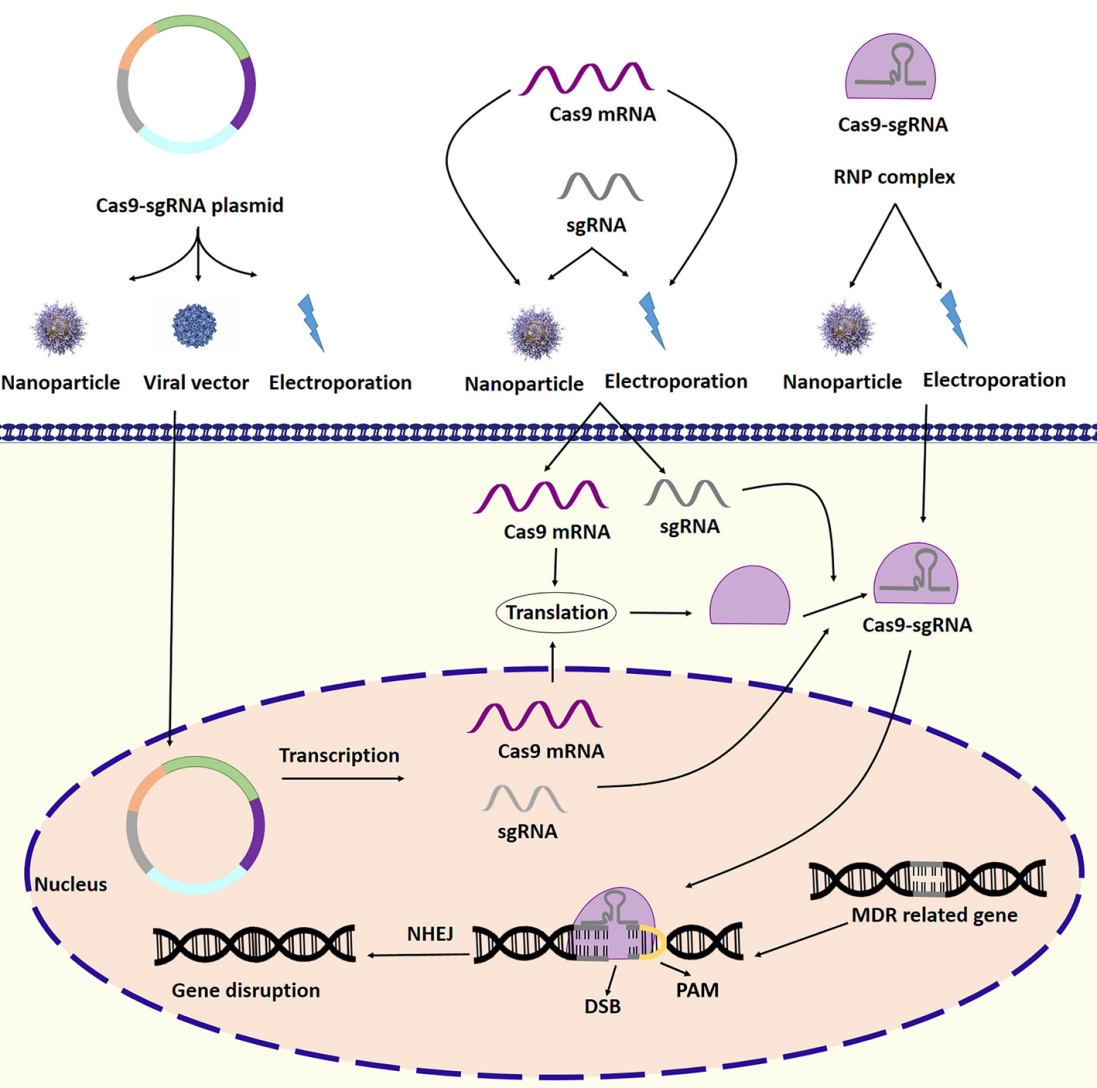
Targeting an MDR-related gene by CRISPR/Cas9 system. Specific sg RNA is designed and produced to target MDR-related gene. The CRISPR system can be transferred into the cell in plasmid, mRNA, and ribonucleoprotein (RNP) complex formats. The use of viral vectors, nanoparticles, and electroporation are among the methods used to deliver the CRISPR/Cas9 system into the cell. In plasmid format, transcription and translation are required to create the sgRNA–Cas9 complex. sgRNA can direct Cas9 to the target gene, and Cas9 generates double-strand break (DBS). The NHEJ repair system then ligates the broken ends. The result of this process is disruption of the target MDR-related gene

In bacteria, the CRISPR/Cas9 system acts like an RNA-based immune system. This system can be used to edit genes in eukaryotic cells, including MDR-related genes. For this purpose, single guide RNA (sgRNA) can be designed to complement the desired sequence and enter the target cell along with Cas9, an endonuclease. sgRNA can guide Cas9 to the target sequence, and Cas9 can cause a double-strand break in the target sequence. In this way, it is possible to delete or insert the desired sequence in to genes [80]. Figure 2 illustrates an example of targeting an MDR-related gene using CRISPR/Cas9 technology. As mentioned in the previous sections, drug resistance is one of the main barriers to the effective treatment of cancers. In recent years, some studies on the drug resistance of cancer cells have focused on the application of CRISPR/Cas9 technology in overcoming drug resistance. These studies have reported very interesting results that have raised hopes for overcoming drug resistance and effective cancer treatment. As mentioned in the previous sections, increasing the expression of ABC family transporters and enhancing the efflux of anticancer drugs are among the most important reasons for drug resistance in cancer cells.

In one of the studies on KBV200 and HCT-8/V cancer cells, it was shown that targeting and knocking out the *ABCB1* gene using CRISPR/Cas9 technology significantly increased the accumulation of doxorubicin (DOX) inside the cells and significantly enhances chemosensitivity [81]. In another study of DOX-resistant breast cancer cells, the *MDR1* gene was targeted and downregulated using the CRISPR/Cas9 system. The results of this study showed that the cytotoxicity of DOX is significantly increased in resistant breast cancer cells treated with Cas9-sgRNA plasmid. Flow cytometric analysis performed in this study showed that targeting MDR1 using CRISPR/Cas9 system in DOX-resistant breast cancer cells increased drug accumulation within the cell and quadrupled drug uptake compared with untreated cells [82]. In another study of ovarian cancer cells, three sgRNAs were designed and used to target the fourth and fifth exons of the *ABCB1* gene. The results of this study showed that the CRISPR/Cas9 system can significantly attenuate the expression of the *ABCB1* gene. In addition, the results of the MTT assay showed that the susceptibility of ovarian cancer cells to doxorubicin was significantly increased [83]. The results of a meta-analysis also showed that targeting the *ABCB1* gene using CRISPR/Cas9 technology could attenuate doxorubicin resistance in drug-resistant osteosarcoma cells [84]. As mentioned in the previous sections, ABC family transporters play a very important role in drug resistance in cancer cells, and increased expression of these transporters is one of the reasons for the failure of chemotherapy. The results mentioned above show that targeting these transporters using CRISPR/Cas9 technology can weaken drug resistance in various cancers. Glutathione *S*-transferase and glutathione are involved in drug detoxification and chemoresistance of cancer cells. Glutathione *S*-transferase may be involved in chemoresistance by enhancing the conjugation of chemotherapy drugs with glutathione, increasing the detoxification of chemotherapy drugs, and attenuating apoptosis [27]. One study showed that knocking out glutathione* S*-transferase omega 1 (GSTO1) in colorectal cancer cells using the CRISPR/Cas9 system could enhance the cytotoxicity of chemotherapy drugs, including cisplatin and oxaliplatin [85]. As mentioned above, strengthening DNA repair could be another mechanism of drug resistance in cancer cells. Cyclin-dependent kinases (CDKs) are very important in this regard, and their role in the drug resistance of cancer cells has been confirmed. CDKs are kinase enzymes involved in regulating the cell cycle, proliferation, and DNA repair [86, 87].

Studies have shown that the expression of these enzymes increases in cancers, and inhibitors of some of these enzymes have very promising results in the treatment of cancer. For example, palbociclib is a CDK4/6 inhibitor that received US Food and Drug Administration (FDA) approval as a breast cancer treatment drug [88]. However, there is also the problem of drug resistance to palbociclib, which appears to be related to CDK6 expression. One study on palbociclib-resistant breast cancer cells showed that CDK6 expression is increased. The results of this study also showed that knocking out CDK6 using CRISPR/Cas9 technology could increase palbociclib sensitivity and reduce the survival of cancer cells after palbociclib treatment [89]. CDK11 also appears to be involved in the drug resistance of cancer cells. A study of ovarian cancer cells showed that CDK11 was involved in enhancing proliferation, attenuating apoptosis, and resistance to paclitaxel [90]. Another study of osteosarcoma cells showed that CDK11 could be targeted and knocked out by designing sg RNA with the TCCGAGACATTTGCTGGGGTGG sequence and using CRISPR/Cas9 technology. In this study, it was shown that knocking out CDK11 using CRISPR/Cas9 technology significantly increased cell death and attenuated the invasiveness of cancer cells [91]. BRCA1 is a well-known and very important tumor suppressor involved in DNA repair [92]. BRCA1 mutations incidence is about 1–7% in breast cancers and ovarian cancers, independently from age and familial history [93]. BRCA1 appears to attenuate the effects of some chemotherapeutic drugs by amplifying DNA repair pathways [94]. These drugs kill cancer cells by inducing DNA damage. High expression of BRCA1 is associated with chemoresistance in cancer cells [95]. BRCA1 mutations (BRCA1m) are highly heterogeneous and difficult to target. However, targeting PARP1, which is the synthetic lethality partner of the *BRCA1* gene, is possible and can enhance chemosensitivity. A study of triple-negative breast cancer cells with mBRCA1 showed that targeting PARP1 using the CRISPR/Cas9 system could increase sensitivity to chemotherapy drugs, including doxorubicin, gemcitabine, and docetaxel, and a lower dose of these drugs is required to achieve therapeutic efficacy [96]. Therefore, a combination of PARP1 inhibitors, CRISPR/Cas9 technology, and chemotherapy may be helpful in the effective treatment of triple-negative breast cancer with mBRCA1. In another study of breast cancer cells, a CRISPR/Cas9 system was designed to target exon 12 HER2 and induce mutations. The results of this study showed that concurrent use of CRISPR/Cas9 technology and PARP inhibitors has a significant inhibitory effect on the growth of cancer cells [97]. Undoubtedly, the simultaneous use of CRISPR/Cas9 technology and DNA repair inhibitors will be of interest in future studies on the treatment of cancers.

RECQL4 helicase is another protein involved in DNA repair and drug resistance. It appears that this protein can also enhance the expression of MDR1 (ABCB1) [98, 99]. A recent study on glioma cells showed that targeting and knocking out RECQL4 using the CRISPR/Cas9 technique could enhance the toxicity of temozolomide (TMZ) on glioma cells so that levels of DNA damage and apoptosis markers, including phospho-H2AX, PARP-1, and cleaved caspases 3 and 7, are significantly increased [98]. Therefore, targeting this protein using CRISPR/Cas9 technology can also be considered as a therapeutic approach to overcome drug resistance in cancers. A significant number of proteins and signaling pathways are involved in the drug resistance of cancer cells. Recent studies have shown that targeting some of these proteins and signaling pathways using CRISPR/Cas9 technology has a significant effect on attenuating drug resistance. Various studies have shown that mutations in oncogenes and tumor suppressors such as K-Ras and P53 play an important role in the drug resistance of cancer cells. These mutations can cause significant changes in associated cellular signaling pathways leading to attenuated apoptosis, enhanced expression of ABC family transporter, and enhanced DNA repair [100–104]. In addition, many important receptors involved in cell growth and proliferation, various components of the signaling pathways associated with them, and a large number of proteins involved in proliferation and apoptosis may undergo mutations leading to enhanced proliferation and drug resistance. Recent findings indicated that targeting oncogenes and mutated tumor suppressors by CRISPR/Cas9 system is possible and has promising effects in overcoming drug resistance in cancer cells. Mutations in K-Ras are known to be among the most important causes of development, progression, drug resistance, and treatment failure in cancers, including colorectal cancer [105, 106]. In an interesting study performed on colorectal cancer (CRC) cells, KRAS gene editing was effectively performed by designing a suitable sgRNA, using the CRISPR/Cas9 system, and using nanotechnology for efficient delivery of the CRISPR/Cas9 system. The results of this study showed that targeting K-Ras using CRISPR/Cas9 technology can significantly enhance the apoptotic effects of cetuximab. The findings also showed that the use of CRISPR/Cas9 to target K-Ras in mice with KRAS-mutated CRC significantly reduced tumor size and enhanced the effect of cetuximab in the induction of apoptosis [107]. It should be noted that cetuximab is an EGFR inhibitor used to treat colorectal cancer with unmutated K-Ras [108].

However, according to the above-mentioned findings, with the help of CRISPR/Cas9 technology and targeting K-Ras, this drug may also be used in colorectal cancer with mutated K-Ras. Undoubtedly, many studies should be done in this field. Mutation in p53 is another event that may occur in cancers and appears to be involved in drug resistance [109]. It appears that knocking out the mutant TP53 in osteosarcoma cells using CRISPR/Cas9 technology could increase sensitivity to doxorubicin and attenuate the expression of anti-apoptotic proteins, including Bcl-2 and survivin [110]. The epidermal growth factor receptor (EGFR) is receptor tyrosine kinase (RTK), which acts as a receptor for members of the epidermal growth factor family. Under normal conditions, this receptor is involved in the development and homeostasis of epithelial tissue, but an increased expression, which may occur because of various mutations, plays an important role in tumorigenesis and drug resistance. Mutations in EGFR are associated with various cancers such as lung cancer and glioblastoma [111, 112]. EGFR inhibitors as a therapeutic approach for the treatment of EGFR-mutant non-small-cell lung cancer have shown positive results; however, there is a problem of drug resistance [113]. In a very interesting study of non-small-cell lung cancer cells, the CRISPR/Cas9 system was designed to selectively target EGFR with a single-nucleotide missense mutation (CTG > CGG). In this study, cancer cells were cotreated by sgRNA-EGFR and Cas9 using adenoviral vectors. This study showed that disruption of mutated EGFR is well performed using CRISPR/Cas9 technology, and this disruption leads to enhancement of cell death. In in vivo examination, a significant decrease in tumor size and an increase in survival rate were also reported in mice [114]. In another study on renal cancer cells, two sgRNA and HDR templates specific for the incision regions were used to target exon 2 and knock out EGFR using CRISPR/Cas9 technology. The results of this study showed that the combination of sunitinib and knocking out EGFR using CRISPR/Cas9 technology has a significant effect on attenuating cancer cell proliferation, while the effect of sunitinib on cancer cells with wild EGFR is less [115]. It should be noted that sunitinib is an FDA-approved drug used to treat renal cell carcinoma. It targets vascular endothelial growth factor (VEGFRs) and platelet-derived growth factor (PDGF-Rs) receptors [116]. Another important protein that is overexpressed in various types of cancer is the urokinase plasminogen activator receptor (uPAR). This protein is a glycosyl-phosphatidyl inositol (GPI)-linked membrane receptor whose main function is focused on the proteolytic activity of urokinase. Urokinase is involved in the breakdown of extracellular matrix (ECM) compounds. uPAR is involved in enhancing EMT and stemness, invasion, metastasis, and drug resistance of cancer cells [117, 118].

In one study of multidrug-resistant cancer cells (HCT8/T and KBV200), the *uPAR* gene was knocked out effectively using CRISPR/Cas9 technology. The results of the MTT test in this study showed that, in cancer cells with knocked out uPAR, IC_50_ of chemotherapy drugs, including 5-FU, cisplatin, docetaxel, and doxorubicin, was significantly reduced, indicating drug resistance attenuation and enhancement of cancer cells’ chemosensitivity [119]. Increased expression of mucin glycoproteins, such as MUC4, has been observed in several cancers, and the overexpression of these glycoproteins appears to be associated with drug resistance [120]. A study on pancreatic cancer cells has shown that MUC4 is involved in the resistance to gemcitabine, a common chemotherapy drug used to treat pancreatic cancer [121]. It has recently been shown that knocking out MUC4 using CRISPR/Cas9 technology can significantly increase the sensitivity of pancreatic ductal adenocarcinoma cells to gemcitabine [122]. Since this cancer is highly lethal, more studies are needed, and it is possible that, using CRISPR/Cas9 technology, chemoresistance can be attenuated in this cancer. Topoisomerase 2 (TOP2) is an enzyme involved in the elimination of DNA topological entanglements during processes such as chromosome condensation and replication through the induction of transient double-strand breaks [123]. Studies have shown that in etoposide-resistant leukemia cells there are two isoforms of this enzyme, TOP2α/170 and TOP2α/90, which appear to be the product of alternative RNA processing. TOP2α/170 is the target of anticancer drugs, but it appears that the expression of this isoform decreases in etoposide-resistant leukemia cells, while the expression of TOP2α/90 increases significantly [124, 125]. This increased expression may play an important role in drug resistance. In a recent interesting study of etoposide-resistant leukemia cells, the CRISPR/Cas9 system was designed to target the exon 19/intron 19 5′ splice site and induce mutations as GAG//GTAA AC → GAG//GTAA GT. The results of this study showed that this gene editing significantly reduced the expression of TOP2α/90 and increased the expression of TOP2α/170, which led to increased sensitivity to etoposide [125]. In addition, studies on chronic myeloid leukemia cells have shown that targeting and disrupting the well-known *BCR*/*ABL* oncogene is also possible using CRISPR/Cas9 gene editing and can lead to attenuated proliferation and enhanced apoptosis in imatinib-resistant leukemia cells [126]. Therefore, it may be possible to overcome the problem of drug resistance in leukemia by using CRISPR/Cas9 technology. Another protein that appears to be involved in the drug resistance of cancer cells is RUNX1. For example, a study of ovarian cancer cells has shown that this protein, along with forkhead box O3 (FOXO3a) and hyperactive insulin-like growth factor-1-receptor (IGF1R), plays a very important role in the drug resistance of ovarian cancer cells [127].

It has recently been shown that knocking out RUNX1 using CRISPR/Cas9 gene editing is possible and may potentiate the apoptotic effects of cisplatin on ovarian cancer cells [128]. Various studies have shown that some signaling pathways can be targeted using CRISPR/Cas9 technology and can lead to attenuation of drug resistance in cancer cells. One of the most important of these pathways is the PI3K/AKT signaling pathway, which is involved in enhancing proliferation, attenuating apoptosis, and enhancing drug resistance [129, 130]. In one study, genes encoding PI3K-110α and PI3K-110β (Pik3ca and Pik3cb) were targeted and knocked out using the CRISPR/Cas9 system. The study showed that targeting P110α and P110β using CRISPR/Cas9 technology could reduce the expression of ABCB1(P-gp) and ABCG2 (BCRP), and partially enhance colchicine and paclitaxel sensitivity in epidermoid carcinoma and lung cancer cells [131]. The relationship between the PI3K/AKT signaling pathway, programmed cell death ligand 1 (PD-L1), and drug resistance is also very interesting. PD-L1 is another protein that appears to be upregulated in drug-resistant cancer cells. This protein may be involved in drug resistance by enhancing the PI3K/AKT signaling pathway. In addition, it seems to play an important role in suppressing antitumor immunity [132, 133]. The results of this study showed that knocking out PDL-1 using CRISPR/Cas9 technology could reduce the IC_50_ of doxorubicin and paclitaxel in osteosarcoma cells, indicating enhanced chemosensitivity in these cells [133]. In “Advances in the study of drug resistance inhibition to enhance chemosensitivity in cancer” section, we discussed the inhibition of NRF2 signaling as an approach to attenuating drug resistance. One study showed that the function of the *NFE2L2* gene, which encodes NRF2, could be disrupted using CRISPR/Cas9 technology, which leads to increased sensitivity of lung cancer cells to cisplatin, vinorelbine, and carboplatin [134]. As mentioned in the previous sections, cancer stem cells have significant resistance to chemotherapy. In fact, one of the main reasons for cancer recurrence and failure of chemotherapy is the presence of cancer stem cells. Some studies have shown that targeting stem cell markers and attenuating stemness using the CRISPR/Cas9 gene-editing technique can help attenuate the drug resistance of cancer cells. In one of these studies, which was performed on prostate cancer cells, NANOG1 and NANOGP8 were effectively knocked out by designing appropriate sgRNAs and using the CRISPR/Cas9 gene-editing technique. The MTT test results of this study showed that sensitivity to docetaxel was significantly increased in NANOG1- and NANOP8-knockout cells [135].

A number of other studies focusing on CD44 have also reported promising results. CD44 is considered a marker of drug resistance and a surface marker of cancer stem cells [136]. This glycoprotein acts as a receptor for hyaluronic acid, which is a component of the extracellular matrix. Studies on ovarian cancer cells have shown that CD44 expression is increased in drug-resistant cancer cells and that CD44 appears to play an important role in paclitaxel resistance [137]. CD44 appears to be involved in drug resistance of cancer cells by various mechanisms, including attenuation of ubiquitination and degradation of ABCB1, enhancement of expression of anti-apoptotic proteins, including Bcl-xL, and regulation of glucose metabolism, including enhancement of glycolysis and pentose phosphate pathway [136, 138, 139]. A study of drug-resistant osteosarcoma cells showed that targeting CD44 using CRISPR/Cas9 technology reduced ABCB1 expression and significantly increased doxorubicin sensitivity [140]. Another study of hepatocellular carcinoma cells also showed that knocking out CD44 using CRISPR/Cas9 technology could increase sensitivity to sorafenib and 5-FU [141]. In addition, the results of a meta-analysis showed that knocking out CD44 using the CRISPR/Cas9 system could inhibit invasion, metastasis, and spheroid formation in osteosarcoma cells [142]. Therefore, targeting CD44 using CRISPR/Cas9 technology can be further studied as an effective approach for cancer treatment. As noted above, the association of miRNAs and lncRNAs with drug resistance of cancers is also very interesting, and some of them seem to be considered as therapeutic targets. Among these noncoding RNAs, the miR-371/372/373 cluster appears to have oncogenic properties and be involved in drug resistance. A recent study on oral carcinoma cells showed that the miR-371/372/373 cluster could be removed using CRISPR/Cas9 technology. The results of this study showed that this deletion significantly enhances the apoptotic effects of cisplatin so that, in deleted subclones, the population of late apoptosis increases significantly after 48 h of treatment with 15 μM cisplatin [143]. One study showed that, using CRISPR/Cas9 technology and lentiviral vector, the expression of miR-21, a well-known oncomiR, in ovarian cancer cells could be inhibited. The results of this study showed that inhibition of miR-21 expression using the CRISPR/Cas9 technique could potentiate the apoptotic effects of paclitaxel and attenuate EMT in ovarian cancer cells [144]. “Advances in the study of drug resistance inhibition to enhance chemosensitivity in cancer” section discussed the role of lncRNA MALAT1 in doxorubicin resistance in breast cancer cells. One study showed that deletion of the lncRNA gene promoter in triple-negative breast cancer cells using the CRISPR/Cas9 technique could enhance doxorubicin and paclitaxel sensitivity [145].

These results suggest that targeting miRNAs and lncRNAs involved in cancer drug resistance using CRISPR/Cas9 technology may be an interesting approach to overcoming drug resistance in cancers and needs to be further studied. Applications of CRISPR/Cas9 gene editing technology are not limited to targeting human proteins. Studies have shown that oncoproteins encoded by the genome of oncogenic viruses can also be targeted using this technique. Undoubtedly, one of the most important oncogenic viruses is human papillomavirus (HPV), which is considered the etiological agent of cervical cancer. HPV16 and HPV18 appear to be responsible for about 70% of precancerous lesions and cervical cancers [146]. Studies have shown that viral oncoproteins E6 and E7 are involved in metastasis and drug resistance of cervical cancer [146, 147]. Studies have shown that disruption of *E6* and *E7* genes, which encode E6 is possible using the CRISPR/Cas9 technique. One study in HPV16-positive cervical cancer cells showed that disruption of the *E7* gene using the CRISPR/Cas9 system could enhance apoptosis, inhibit growth, and increase pRb expression [148]. Another study showed that disruption of HPV16 E6/E7 using the CRISPR/Cas9 technique enhances the effect of cisplatin on growth inhibition and induction of apoptosis in cervical cancer cells and xenograft mice models [149]. Another study showed that knocking out HPV18 E6 using CRISPR/Cas9 technology could potentiate the effect of cisplatin on attenuating proliferation and enhancing apoptosis in cervical cancer cells [150] (Table 1). All of the above suggests that CRISPR/Cas9 gene editing can be useful in overcoming drug resistance in various cancers and that different mechanisms of drug resistance can be targeted using this technique. Given the many studies that are currently underway, it is hoped that, by finding solutions to overcome the limitations of the CRISPR/Cas9 technique, the way will be opened for the clinical use of this technology to overcome drug resistance in cancers.

**Table 1:** The target genes and effectiveness of CRISPR/Cas9 technology in overcoming drug resistance

Drug	CRISPR/Cas9-targeted gene	Type of cancer cell	Total effect	References
Doxorubicin	*ABCB1*	Epidermoid carcinoma and colorectal cancer	Accumulation of drug in the cancer cells and increasing chemosensitivity	[81]
Doxorubicin	*MDR1*	Breast cancer	Accumulation and uptake of drug in the cells and increasing the cytotoxicity of drug	[82]
Doxorubicin	*ABCB1*	Ovarian cancer	Increasing chemosensitivity	[83]
Doxorubicin	*ABCB1*	Osteosarcoma	Decreasing doxorubicin resistance	[84]
Cisplatin and oxaliplatin	*GSTO1*	Colorectal cancer	Increasing the cytotoxicity of drugs	[85]
Palbociclib	*CDK6*	Breast cancer	Increasing cancer cell sensitivity to anticancer drug and reducing cancer cell survival	[89]
Doxorubicin, gemcitabine, and docetaxel	*PARP1*	Triple-negative breast cancer	Increasing chemosensitivity and the inhibition of cancer cell growth	[96, 97]
Temozolomide	*RECQL4*	Glioma	Increasing DNA damage and apoptotic markers	[98]
Cetuximab	*KRAS*	Colorectal cancer	Increasing apoptosis induction and reducing tumor size	[106]
Doxorubicin	*TP53*	Osteosarcoma	Reducing anti-apoptotic proteins	[110]
Sunitinib	*EGFR*	Renal cancer	Reducing cancer cell proliferation	[115]
5-FU, cisplatin, docetaxel, and doxorubicin	*uPAR*	HCT8/T and KBV200	Reducing IC_50_ and attenuating drug resistance	[119]
Gemcitabine	*MUC4*	Pancreatic cancer	Increasing chemosensitivity	[122]
Etoposide	*TOP2α/90*↓ *TOP2α/170*↑	Etoposide-resistant leukemia cells	Increasing chemosensitivity	[125]
Imatinib	*BCR*/*ABL*	Chronic myeloid leukemia	Reducing cell proliferation and increasing apoptosis in resistant cells	[126]
Cisplatin	*RUNX1*	Ovarian cancer	Increasing chemosensitivity	[128]
Colchicine and paclitaxel	*PI3K-110α* and *PI3K-110β* (*Pik3ca* and *Pik3cb*)	Epidermoid carcinoma and lung cancer	Increasing chemosensitivity	[131]
Cisplatin, vinorelbine, and carboplatin	*NFE2L2*	Lung cancer	Increasing chemosensitivity	[134]
Doxorubicin and paclitaxel	*PDL-1*	Osteosarcoma	Reducing IC_50_ and increasing chemosensitivity	[133]
Docetaxel	*NANOG1* and *NANOGP8*	Prostate cancer	Increasing chemosensitivity	[135]
Doxorubicin	*CD44*	Osteosarcoma	Reducing ABCB1 expression and increasing chemosensitivity	[140]
Sorafenib and 5-FU	*CD44*	Hepatocellular carcinoma	increasing chemosensitivity	[141]
Cisplatin	*miR-371*/*372*/*373*/*373* cluster	Oral carcinoma	Induction of apoptotic effect of drug and increasing chemosensitivity	[143]
Paclitaxel	*miR-21*	Ovarian cancer	Increasing chemosensitivity Attenuating EMT	[144]
Doxorubicin and paclitaxel	lncRNA *MALAT1*	Triple-negative breast cancer	Increasing chemosensitivity	[145]
Cisplatin	*HPV16* *E6*/*E7*	Cervical cancer cells and xenograft mouse models	Inducing apoptosis and inhibiting cell growth as well as reducing cell proliferation	[149, 150]

## Application of CRISPR/Cas9 system in identification of drug-resistance-related genes and understanding drug resistance mechanisms

CRISPR/Cas9 technology can also be used to identify drug resistance mechanisms in cancer cells. In recent years, several studies using CRISPR/Cas9 technology have identified some important genes associated with drug resistance in cancer. In this section, we will review the findings of some of these studies. Some preclinical studies using CRISPR/Cas9 technology have highlighted the importance of some genes, including *SLFN11*, *APE1*, *RSF1*, and *CDK5*, in cancer drug resistance [151]. Schlafen 11 (SLFN11) is a DNA/RNA helicase and is involved in blocking replication and stimulating cell death [152]. SLFN11 appears to enhance cancer cells’ sensitivity to a wide range of anticancer drugs, including platinum derivatives, DNA synthesis inhibitors such as gemcitabine, and topoisomerase inhibitors such as irinotecan [153]. One study found that knocking out the *SLFN11* gene using the CRISPR/Cas9 technique could induce resistance to talazoparib, a PARP inhibitor in small-cell lung cancer cells, which indicates the role of SLFN11 in attenuating drug resistance and suggests that SLFN11 expression may be a predictive marker of response to the PARP inhibitor in cancer cells [154]. The mechanism of action of SLFN11 in enhancing sensitivity to PARP inhibitors was elucidated in another study using CRISPR/Cas9 technology. In this study, SLFN11-deleted cells were generated using CRISPR/Cas9 technology. This study showed that, after treatment with talazoparib, SLFN11-deleted cells had attenuated inhibition of replication at 24 h and reached the G2 phase at 48 h, and the increase in the percentage of apoptotic cells was not significant. The study suggested that SLFN11 may induce apoptosis and enhance the sensitivity of cancer cells to PARP inhibitors by enhancing S-phase arrest [155]. APE1 (apurinic/apyrimidyl endonuclease 1) is considered a rate-limiting enzyme in the BER DNA repair system. In one study using CRISPR/Cas9 technology, this enzyme was found to be involved in attenuating olaparib resistance in triple-negative breast cancer. The results of this study showed that knocking out the *APE1* gene in triple-negative breast cancer cells could significantly increase the IC_50_ of olaparib, suggesting that APE1 deletion may be associated with olaparib resistance [156]. Remodeling and spacing factor 1 (RSF1) is an important protein mainly involved in chromosome stabilization, attenuation of transcription of some oncogenes, and enhancement of DNA repair. Increased expression of this protein appears to be associated with drug resistance in cancers [157].

A study on lung cancer cells showed that silencing RSF-1 using CRISPR/Cas9 technology could potentiate the apoptotic and antitumor effects of paclitaxel. The results of this study showed that knocking out RSF-1 also enhances the effects of paclitaxel on reducing tumor volume and weight in xenograft mice. The results of this study suggest that RSF-1 may be involved in enhancing drug resistance in cancer cells by enhancing NFKB signaling [158]. In the previous section, we mentioned CDKs and their role in regulating the cell cycle, proliferation, and DNA repair. Unlike the CDKs mentioned in the previous section, which are known for their role in regulating the cell cycle, CDK5 is considered an atypical CDK and is known for its functions in the central nervous system (CNS) [159]. CDK5 plays an important role in the regulation of neuronal migration during CNS development and is also involved in other events such as drug addiction and synaptic plasticity [160]. However, various studies have shown that CDK5 is involved in tumorigenesis and the progression of a wide variety of cancers and may also be associated with drug resistance [159]. One study using CRISPR/Cas9 technology to silence CDK5 clearly showed that knocking down CDK5 could potentiate the effects of sorafenib on attenuating the proliferation and survival of hepatocellular carcinoma cells. The results of this study elucidated the mechanism of the effect of CDK5 knockdown on enhancing the effectiveness of sorafenib and showed that CDK5 knockdown can attenuate the induction of EGFR and AKT phosphorylation following treatment with sorafenib. In addition, the results of this study showed that CDK5 knockdown inhibited the increase in EGFR levels at the cell surface following sorafenib treatment. It should be noted that these events and, in general, the compensatory activation of EGFR signaling are key mechanisms of resistance to sorafenib [161, 162]. AT-rich interactive domain 1A (*ARID1A*), aurora kinase B (*Aurora-B*), α-thalassemia/mental retardation syndrome X-linked (*ATRX*), and baculoviral IAP repeat-containing 5 (*BIRC5*) are also among the genes involved in cancer drug resistance. The mechanism of action of these genes in drug resistance has been elucidated by CRISPR/Cas9 technology. Progesterone resistance is one of the main barriers to the treatment of endometrial cancer with medroxyprogesterone (MPA). One study using CRISPR/Cas9 technology identified interesting aspects of the mechanism of progesterone resistance in endometrial cancer cells. The results of this study showed that knocking out the *ARID1A* gene, a tumor suppressor, could downregulate progesterone receptor B (PRB), enhance AKT phosphorylation, and attenuate MPA sensitivity in endometrial cancer cells [163].

These results suggest a mechanism by which ARID1A may regulate PRB expression and play an important role in MPA sensitivity in endometrial cancer cells. Aurora-B is involved in the regulation of mitotic division, and its increased expression appears to be associated with enhanced resistance to cisplatin and paclitaxel in non-small-cell lung cancer cells and decreased overall survival in patients. Knocking out this gene using the CRISPR/Cas9 system had promising results in enhancing chemo-sensitivity and increasing p53 expression. It appears that Aurora-B can attenuate apoptosis and enhance drug resistance in cancer cells by attenuating the p53-dependent DNA damage response [164]. ATRX plays a variety of roles in the cell, including maintaining the structural integrity of the telomere, replicating DNA, and repairing DNA [165–167]. A study of glioma cells showed that knocking out the *ATRX* gene using the CRISPR/Cas9 system could significantly enhance the apoptotic effects of TMZ. The results of this study showed that knocking out this gene could reduce the availability of histone H3K9me3 and attenuate ATM-dependent DNA repair [167]. These findings suggest that ATRX may be involved in drug resistance by enhancing ATM-dependent DNA repair. *BIRC5* is a gene encoding survivin, which is an anti-apoptotic protein [168]. A study of ovarian cancer cells showed that disrupting this gene using the CRISPR/Cas9 system could upregulate cytokeratin-7, an epithelial marker, downregulate mesenchymal markers, including vimentin, snai2, and β-catenin, and enhances sensitivity to paclitaxel [169]. The results of this study suggest that BIRC5 may be involved in drug resistance of cancer cells by enhancing EMT. Identification of various genes involved in drug resistance and their mechanism of action by CRISPR/Cas9 technology, some of which were mentioned above, will undoubtedly pave the way for future studies on these genes as therapeutic targets to overcome drug resistance in cancers. In recent years, the genome-wide CRISPR/Cas9 screen technique has been very helpful in identifying the genes involved in the drug resistance of cancer cells. This method uses a collection of designed sgRNAs or so-called sgRNA libraries. Recently, these libraries have become commercially available. Lentiviral vectors are used to deliver these sgRNA libraries and Cas9, which is one of the most common delivery methods used in CRISPR/Cas9 gene editing. Briefly, a pool of sgRNAs is designed to identify the genes responsible for drug resistance, and then a large population of cells is infected by a library of lentiviral vectors containing these sgRNAs and Cas9. Then drug treatment can be administered. The desired clones are then selected on the basis of phenotypic features, and the DNA is extracted. Then, using next-generation sequencing, it is determined which genes are present in the drug-sensitive and drug-resistant cells and which have been deleted [170, 171].

It should be noted that genome-wide CRISPR knockout screens can also be done as an arrayed screen, which is done using wells. In this method, each well contains a specific sgRNA and does not require next-generation sequencing [170]. In general, using the genome-wide CRISPR knockout screens technique, it is possible to examine a large number of genes in an experiment. A significant number of studies in recent years that have focused on the identification of genes involved in drug resistance have used this technique and reported interesting results. For example, some preclinical studies using the CRISPR screen technique have elucidated the mechanism of action of PBRM1 and SGOL1 in cancer drug resistance. PBRM1 is a SWI/SNF complex subunit. This complex is involved in altering the nucleosome position and controlling chromatin availability and appears to have tumor-suppressive properties [172–174]. One study, performed on lung cancer cells using the CRISPR screen, elucidated the mechanism of action of PBRM1 in resistance to EGFR inhibitors. In this study, it was shown that mutations in this gene may attenuate the effects of EGFR inhibitors by enhancing the continuity of AKT signaling [174]. SGOL1 is a protein involved in the protection of centromeric cohesion during meiotic division, and its knockdown appears to cause chromosomal instability [175, 176]. One study using the CRISPR screening technique and a library consisting of 123,411 sgRNAs identified the *SGOL1* gene as a marker of prognosis in sorafenib-treated patients. The results of this study showed that silencing SGOL1 using the CRISPR/Cas9 system and sgRNA may cause resistance to sorafenib in hepatocellular carcinoma cells. The in vivo results of this study also showed that loss of SGOL1 could attenuate the cytotoxic effect of sorafenib [177]. Identification of genes involved in cancer drug resistance using the genome-wide CRISPR/Cas9 screen technique is not limited to the above examples, and a significant number of genes have been identified using this technique in recent years, which are briefly reviewed below. In one of the recent studies on prostate cancer cells, the genome-wide CRISPR/Cas9 screen showed that 17 genes play a key role in docetaxel resistance, and inhibition of them could increase sensitivity to docetaxel. The results of this study showed that transcription elongation factor A-like 1 (Tceal1) is very important among these genes, and its suppression can increase the effects of the docetaxel in amplifying subG1 cell death and polyploidy [178]. Another study on cervical cancer cells identified 374 genes involved in paclitaxel resistance, including *ABCC9* and *IL37*, using genome-wide CRISPR/Cas9 screen technology [179].

One study on bladder cancer cells identified the importance of the *MSH2* gene and mismatch repair in cisplatin resistance using the genome-wide CRISPR/Cas9 screen. The results of this study showed that the knockdown of this gene may attenuate the apoptotic effects of cisplatin [180]. In a study on gallbladder cancer, the role of elongator complex subunit 5 (ELP5) in gemcitabine sensitivity was identified using the genome-wide CRISPR/Cas9 screen. On the basis of the results of this study, low ELP5 expression following gemcitabine treatment may be associated with poor survival in patients with gallbladder cancer [181]. In another study on renal cell carcinoma, genome-wide CRISPR/Cas9 screen results showed that farnesyltransferase is probably one of the most important factors involved in sunitinib resistance. In this study, farnesyltransferase was knocked down for further investigation using appropriate siRNA, and it was observed that knockdown of this enzyme enhances the apoptotic effects of sunitinib. The results of this study also showed that lonafarnib, a farnesyltransferase inhibitor, could potentiate the antitumor effects of sunitinib [182]. In another study on NRAS-mutant melanoma, genome-wide CRISPR/Cas9 screens were used to identify genes involved in resistance to trametinib, which is a MEK1/2 inhibitor. Genome-wide CRISPR/Cas9 screens showed that Kelch domain-containing F-Box protein 42 (FBXO42), an E3 ubiquitin ligase, plays an important role in trametinib resistance. In addition, the results of this study showed that FBXO42 appears to be involved in the TAK1 signaling pathway. The results of this study suggest a combination therapy with TAK1 inhibitor and trametinib as an effective therapeutic approach for the treatment of NRAS-mutant melanoma [183]. In a study on acute myeloid leukemia (AML) cells, the results of genome-wide CRISPR/Cas9 screens revealed negative regulators of the MAPK and mTORC1 signaling pathways, including LZTR1, NF1, and TSC1 or TSC2, were associated with sorafenib resistance [184]. In another study on AML cells, genes involved in the resistance to TAK-243, a UBA1 inhibitor (ubiquitin-like modifier activating enzyme 1), were studied using genome-wide CRISPR/Cas9 screens. The results of genome-wide CRISPR/Cas9 screens showed that BEN domain-containing protein 3 (BEND3) plays a key role in the efficacy of TAK-243 so that knockout of this protein is associated with attenuation of the effect of TAK-243 on DNA repair response and proteotoxic stress. The results of this study also showed that knocking out this protein, which is involved in chromatin organization regulation, could increase ABCG2 expression and decrease TAK-243 intracellular levels, which may lead to TAK-243 resistance [185].

**Fig. 3:**
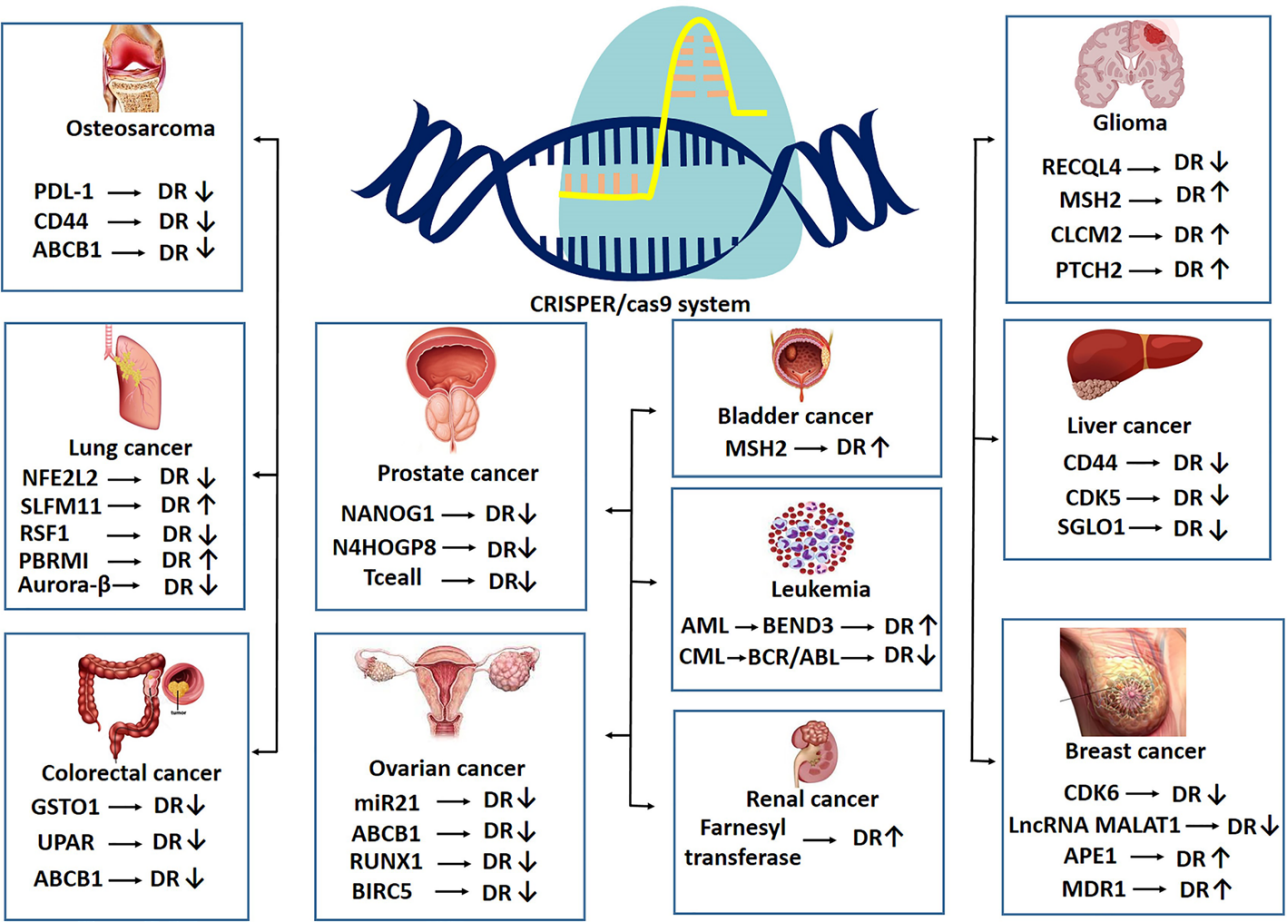
Identified genes involved in drug resistance and effects of CRISPR/Cas9-mediated inhibition on drug resistance. *DR* drug resistance

Another study on k-ras mutated colorectal cancer cells using genome-wide CRISPR/Cas9 screens technology identified the role of the Wnt/B-catenin signaling pathway in resistance to BCL-XL inhibitor ABT-263. The results of this study showed that, in ABT-263-resistant cells, sgRNAs that target positive regulators of WNT signaling are depleted, while sgRNAs that target negative regulators of this signaling pathway are enriched [186]. These results demonstrate the pivotal role of Wnt signaling in resistance to ABT-263 in k-ras mutated colorectal cancer cells. In a study of glioblastoma cells, the key role of a number of genes, including *MSH2*, *CLCA2*, and *PTCH2*, in the resistance to temozolomide (TMZ) was elucidated using genome-wide CRISPR/Cas9 screen technology. These genes are involved in mismatch repair, Wnt signaling pathway inhibition, and sonic hedgehog pathway inhibition, respectively. Further investigation in this study showed that silencing of these genes using siRNA increases cell viability following TMZ treatment, indicating the effect of activation of the Wnt and Sonic Hedgehog signaling pathways on drug resistance in glioblastoma cells [187] (Table 2). In general, and based on the results of the studies mentioned above, genome-wide CRISPR/Cas9 screen technology has revolutionized the identification of the molecular mechanism of drug resistance in cancers. The above-mentioned studies, which have often been published in the last few years, have identified some very important genes in cancer drug resistance by the genome-wide CRISPR/Cas9 screen that could be further studied as therapeutic targets. Some of these genes are summarized in Fig. 3. These very interesting findings promise that, in the not-too-distant future, a revolution in cancer treatment may occur by overcoming the barrier of drug resistance. Undoubtedly, in the coming years, much greater dimensions of drug resistance mechanisms in cancer will be discovered using the genome-wide CRISPR/Cas9 screen, and the course of studies will be much faster.

**Table 2:** Identified genes involved in drug resistance using CRISPR/Cas9 technology

Genes responsible for drug resistance	Type of cancer cell	Possible effects	References
*SLFN11*	Lung cancer	Enhancing S-phase arrest Enhancing apoptosis Attenuating resistance to talazoparib	[154, 155]
*APE1*	Triple-negative breast cancer	Attenuating resistance to olaparib	[156]
*RSF1*	Lung cancer	Enhancing NFKB signaling Enhancing paclitaxel resistance	[158]
*CDK5*	Hepatocellular carcinoma	Enhancing sorafenib resistance	[161]
*ARID1A*	Endometrial cancer	Upregulating PRB Enhancing cancer cell sensitivity to MPA	[163]
*Aurora-B*	Lung cancer	Attenuating the p53-dependent DNA damage response Enhancing resistance to cisplatin and paclitaxel	[164]
*ATRX*	Glioma	Enhancing ATM-dependent DNA repair Enhancing resistance to TMZ	[167]
*BIRC5*	Ovarian cancer	Enhancing EMT Enhancing paclitaxel resistance	[169]
*PBRM1*	Lung cancer	Attenuating AKT signaling Enhancing effectiveness of EGFR inhibitors	[174]
*SGOL1*	Hepatocellular carcinoma	Enhancing cytotoxic effect of sorafenib	[177]
*Tceal1*	Prostate cancer	Increasing cell death, polyploidy, and docetaxel sensitivity might happen after knockout	[178]
*ABCC9* and *IL37*	Cervical cancer	Paclitaxel resistance	[179]
*MSH2*	Bladder cancer	Reducing apoptotic effect of cisplatin might happen after gene knockout	[180]
*ELP5*	Gallbladder cancer	Poor survival might happen after ELP5 knockout and gemcitabine treatment	[181]
*Farnesyltransferase*	Renal cell carcinoma	Sunitinib resistance	[182]
*FBXO42*	NRAS-mutant melanoma	Trametinib resistance	[183]
*LZTR1*, *NF1*, and *TSC1* or *TSC2*	Acute myeloid leukemia	Sorafenib resistance	[184]
*BEND3*	Acute myeloid leukemia	Increasing ABCG2 level, reducing TAK-243 in the cell and drug resistance	[185]
*Wnt*/*B-catenin*	k-ras mutated colorectal cancer	ABT-263 resistance	[186]
*MSH2*, *CLCA2*, and *PTCH2*	Glioblastoma	TMZ resistance	[187]

## Limitation of CRISPR/Cas9 gene editing: is there a long way to the clinic?

Despite all the applications of CRISPR/Cas9 gene-editing technology mentioned in the previous sections, this technique also has limitations that must be eliminated or minimized before clinical application. Of course, discussing the limitations of the CRISPR/Cas9 gene-editing technique requires writing a separate review article. However, in this section, we have tried to give a very brief overview of the most important of these limitations. Undoubtedly, off-target mutagenesis is one of the most important limitations of CRISPR/Cas9 gene editing. Certain sequences of DNA are targeted in this method. However, sometimes sgRNA creates off-target mutations by affecting other regions that resemble the target sequence, which may disrupt the normal function of the gene and cause genomic instability. The frequency of off-target mutations seems to be higher than 50%. Therefore, overcoming this limitation may be a big step toward the clinical application of CRISPR/Cas9 gene-editing technology [188]. The off-target effect can be reduced with the correct design of sgRNA. For this purpose, platforms such as CHOPCHOP, E-CRISP, and CRISPR-ERA have been developed. Employing these computational tools, it is possible to design suitable and specific sgRNAs [189]. Another way to reduce the off-target effect is to use D10A-mutated Cas9. This variant of Cas9 creates single-strand breaks instead of double-strand breaks, and it seems that using it with a pair of sgRNA can reduce the off-target effect significantly. An off-target single-strand break can be repaired by local enzymes [190]. Other variants of Cas9 have also been developed, including SpCas9-HF1 and eSpCas9 (1.1), which appear to significantly reduce the off-target effect [190]. As mentioned in the previous sections, the identification of the PAM region near the target site by Cas9 is a prerequisite for a double-strand break. In fact, this sequence acts as a binding signal for Cas9. This can be considered a limitation for CRISPR/Cas9 gene editing. SpCas9, commonly used for gene editing, PAM site is 5′NGG3′ (N can be any nucleotide). To overcome this limitation and expand the gene target window, various variants of Sp Cas9 have been developed, including SpCas9-NG and xCas9 [191]. DSBs generated by the CRISPR/Cas9 system may activate p53 and induction of apoptosis in the cell. To overcome this limitation, the use of Cas9 variants with the ability to create single-strand breaks has been suggested [191].

A serious limitation to the clinical application of CRISPR/Cas9 gene editing is the possibility of the presence of anti-SpCas9 antibodies, which has been reported by some studies, and studies are needed to find appropriate solutions to overcome this limitation [191, 192]. A study on *Campylobacter jejuni* Cas9 (CjCas9) can be helpful in this regard [191]. CRISPR/Cas9 system delivery should also be further studied. The current common method for delivering this system is AAV vectors. There are limitations, such as stimulating an immune response and increased off-target mutations due to the long-term expression of system components in this delivery method. The use of nonviral vectors such as nanoparticles has been suggested to limit the stimulation of the immune response. In vivo delivery also has limitations such as degradation by certain enzymes and immune cells [191, 193, 194]. Therefore, before the clinical application of this technology to combat the problem of drug resistance of cancers, many studies should be conducted to find appropriate solutions to overcome all the limitations mentioned above. It is hoped that overcoming these limitations will pave the way for the clinical application of CRISPR/Cas9 gene-editing technology in the future (Table 3).

**Table 3:** Some limitations and solutions in the application of CRISPR/Cas9 gene editing

Limitations	Solutions	References
Off-target mutagenesis	Correct design of sgRNA D10A-mutated Cas9 SpCas9-HF1 and eSpCas9	[188–190]
Identification of the PAM region	SpCas9-NG and xCas9 variants	[191]
P53 activation and apoptosis	Cas9 variants with the ability to create single-strand breaks	[191]
Anti-SpCas9 antibodies	*Campylobacter jejuni* Cas9 (CjCas9)	[191, 192]
Stimulation of immune responses in delivery by viral vector	Using other delivery method such as nanoparticle	[193]

## Conclusion and future direction

CRISPR/Cas9 gene editing can be used as an effective approach to overcome the challenge of drug resistance in cancers. Using this technology, it is possible to target and knock out the genes of ABC family transporters, which leads to the attenuation of anticancer drug efflux. Using the CRISPR/Cas9 technique, it is possible to target various components involved in DNA repair such as RECQL4 helicase and PARP1, which can attenuate the DNA repair ability and enhance the effectiveness of some anticancer drugs such as temozolomide and doxorubicin. In addition, this technique can be used to target mutated EGFR, K-Ras, mutant TP53, uPAR, BCR/ABL, and some other oncogenes and signaling pathways involved in the drug resistance of cancer cells. Using the CRISPR/Cas9 technique, it is possible to target stem cell markers such as NANOG1, NANOGP8, and CD44, which can lead to a significant attenuation of drug resistance in cancer cells. In addition to these, genome-wide CRISPR/Cas9 screen has helped to shed light on more aspects of drug resistance mechanisms in cancer cells, and significant information has been added to current knowledge in recent years. The CRISPR/Cas9 technique has limitations, including off-target mutagenesis, and effective solutions are required to overcome the limitations of this technique.

There are currently several clinical trials to evaluate the effectiveness of CRISPR/Cas9 gene editing in the treatment of cancer. Considering the interesting findings reviewed in this article, designing clinical trials to evaluate the effectiveness of this technique in attenuating drug resistance in cancers can be helpful.

## Data Availability

Not applicable.
